# Vitamin D: Giveth to Those Who Needeth

**DOI:** 10.1002/jbm4.10232

**Published:** 2019-11-14

**Authors:** Paul Lips, John P Bilezikian, Roger Bouillon

**Affiliations:** ^1^ Department of Internal Medicine, Endocrine Section Amsterdam University Medical Center Amsterdam The Netherlands; ^2^ Division of Endocrinology Columbia University College of Physicians and Surgeons New York NY USA; ^3^ Laboratory of Clinical and Experimental Endocrinology, Department of Chronic Diseases Metabolism and Ageing Leuven Catholic University of Leuven Belgium

**Keywords:** VITAMIN D, CALCIUM SUPPLEMENTS, OSTEOPOROSIS, FRACTURES, RICKETS, META ANALYSIS

## Abstract

Severe vitamin D deficiency may cause rickets. While this point is not disputed, the use of vitamin D in the elderly to prevent fractures has been challenged recently by a meta‐analysis of 81 RCTs, suggesting that the effects of vitamin D were trivial. As is true for any review of the literature, the interpretation of a meta‐analysis can be confounded by the choice of publications to include or exclude. Indeed, the authors excluded RCTs with combined vitamin D and calcium supplementation, included futile studies of very short duration, or studies with high bolus doses known to transiently increase fracture risk. The best available data show that calcium and vitamin D supplementation of elderly subjects can decrease the risk of hip and other non‐vertebral fractures, especially in institutionalized subjects or elderly subjects with poor calcium and vitamin D status. Vitamin D deficiency is associated with many chronic diseases. The VIDA and VITAL trials did not show a protective effect on cardiovascular diseases and cancer. The D2d study also did not influence the progress of prediabetes to diabetes. However, the baseline 25OHD concentrations of the majority of the participants of all these trials were essentially normal. Post‐hoc analysis of these studies suggest some possibly beneficial health outcomes in vitamin D deficient subjects. A meta‐analysis suggested that vitamin D could partly prevent upper respiratory infections. Mendelian randomization studies suggest a causal link between lifelong low vitamin D status and multiple sclerosis. A vitamin D supplement in pregnant women may decrease maternal morbidity and improve the health of their offspring. Better‐designed studies are needed to answer all outstanding questions. However, based on all available data, it seems that correction of vitamin D and/or calcium deficiency of infants, pregnant women and elderly subjects can improve their health. © 2019 The Authors. *JBMR Plus* published by Wiley Periodicals, Inc. on behalf of American Society for Bone and Mineral Research.

Vitamin D deficiency causes rickets; a low dose (about 10 μg or 400 IU/d) of vitamin D can prevent it. Severe clinical vitamin D deficiency in adults, known as osteomalacia, has been reported in older patients who sustain hip fractures. Vitamin D deficiency also accelerates the development of osteoporosis.^1^, ^2^ A randomized clinical trial of French nursing home residents demonstrated that daily vitamin D (800 IU) and calcium (1200 mg) supplements could prevent up to 25% of hip fractures and other nonvertebral fractures.^(3)^ During the past several decades, vitamin D nutritional status has also been associated with many chronic extraskeletal diseases. These observations have given rise to beliefs held by some experts and lay people that vitamin D can cure or ameliorate most diseases. Although this would seem to be a fanciful proposition, impressive preclinical data have shown that the vitamin D endocrine system is involved in a wide spectrum of physiological actions, in line with most other ligands that interact with nuclear receptors, such as the thyroid hormone, sex hormones, and glucocorticoids. The vitamin D receptor and its activating enzyme, 1α‐hydroxylase (CYP27B1), are present in many cells and tissues, suggesting not only a classical hormonal role but also a local role in many tissues.^4^, ^5^ After binding to the vitamin D receptor, 1,25‐dihydroxyvitamin D [1,25(OH)_2_D], the active metabolite, can up‐ or downregulate more than 300 genes in mice and humans and as much as 10% of the zebrafish genome.^5^ Systemic or tissue‐specific deletion of the vitamin D receptor or CYP27B1, which represents the most severe form of vitamin D deficiency, worsens many diseases.^5^ Hundreds of epidemiologic studies have associated poor vitamin D status with the most chronic diseases: from cancer to infectious and autoimmune diseases, from dysfunction of skeletal and cardiac muscle to cardiovascular, neurological, and metabolic diseases—ultimately all leading to higher mortality risks. This tantalizing background information generated a plethora of plausible hypotheses, followed by about 3000 NIH TrialNet registered randomized clinical trials (RCTs).

Epidemiologic data, however, are hypothesis‐generating and do not establish causation. Confirmation is needed by RCTs, Mendelian randomization (MR) approaches, and appropriately designed meta‐analyses. In addition, the supposed systemic actions of vitamin D coupled to mean concentrations of 25OHD (about 46 ng/mL) in African peoples, have stimulated the use of vitamin D to match the levels of East Africans who traditionally wear few clothes and are always exposed to the sun.^6^ Further support for the use of high doses (up to 10,000 IU/d) is based on the erroneous idea that a full day of sun exposure generates more than 10,000 IU of vitamin D per day.^7^ An ultraviolet radiation study in older persons at half the minimal erythematous dose on 1000 cm^2^ of the back three times per week led to an increase of serum 25OHD equivalent to 3000 IU/week or 400 IU/d. An extrapolation of these data to the whole body would lead to a dose equivalent of about 5000 IU for whole‐body irradiation at half the minimal erythematous dose.^8^ Even lower daily production was found in a study of Danish women subjected to intensive sun exposure.^9^

The rather exuberant expectation that vitamin D is pervasively important in virtually all human tissues and plays a role in many diseases has been tempered by the Institute of Medicine (IOM), which has set the minimal desirable concentration of serum 25‐hydroxyvitamin D at 50 nmol/L (20 ng/mL) for the general population.^10^ This led to modest revisions in its recommendations for daily nutritional amounts of vitamin D: a range from 400 to 800 IU/d depending on age. This recommendation has been countered by other authoritative bodies such as the Endocrine Society, which has set a higher recommended minimal level at 75 nmol/L (30 ng/mL).^11^ It is important to note that these guidelines are for a normal, healthy population. The IOM, in particular, did not address the possibility that patients with a metabolic bone disease might require higher circulating levels of 25‐hydroxyvitamin D.

Recently, the effects of vitamin D on the aging skeleton have been challenged. Meta‐analyses of randomized clinical trials of vitamin D versus placebo have suggested that the effect of vitamin D, if any, was trivial, and too small to be clinically relevant.^12^, ^13^ In their last meta‐analysis, Bolland and colleagues^(13)^ found that vitamin D did not decrease the incidence of fractures in older persons and concluded that vitamin D should not be prescribed for this purpose. Furthermore, they suggested that the guidelines should be reformulated. An accompanying editorial also suggested, “vitamin D: the end of the story.”^14^ However, this meta‐analysis has generated criticism and more controversy.^15^, ^16^, ^17^ First, the authors did not include clinical trials combining vitamin D with calcium supplements, thus deliberately excluding the most successful trials, eg, those performed in Lyon, France.^3^, ^18^ In addition, they included trials that lasted less than 12 months, too short a period to evaluate an effect of any intervention on fracture incidence. They also included trials with very high annual doses that are now thought to increase fracture incidence.^19^, ^20^ In addition, the meta‐analysis includes many studies that may not have reached adequate final 25OHD concentrations.^21^ However, the authors of this controversial meta‐analysis, while claiming the futility of the use of vitamin D, published independently in 2014^22^, ^23^ that combined vitamin D and calcium supplements can reduce the risks of hip and nonvertebral fractures in the elderly. As most frail older persons get little vitamin D from diet and sun exposure and consume low‐to‐moderate amounts of dairy, they are thus frequently deficient in their regular supply of both calcium and vitamin D. It seems, therefore, reasonable, as we and others have suggested, to propose that most elderly may derive benefit from modest vitamin D and calcium supplements to prevent fractures.^22^ RCTs have shown that vitamin D decreases the incidence of hip fractures and other nonvertebral fractures by about 15%, the effect being greater in the 70‐ to 80+‐year‐olds than in younger 60‐ to 70‐year‐olds, and in the institutionalized rather than in the community‐living elderly, when combined with calcium and a compliance rate of >80%.^5^

Another important issue regards the possible effects of vitamin D on extraskeletal outcomes, as extensively discussed by a large group of experts.^5^ Although epidemiological studies (eg, the Longitudinal Aging Study Amsterdam) have shown many associations, including cardiovascular disease, diabetes, respiratory infection, cancer, autoimmune disease, and depression,^24^, ^25^the results of randomized clinical trials have not been as definitive.

Negative results of many RCTs have been attributed to normal or virtually normal baseline serum 25OHD levels, an inadequate amount of vitamin D in the RCT, reverse causation, or underpowered studies. Recently, the results of several mega‐trials have been published. Illustrative of these difficulties, the Vitamin D Assessment (ViDA) Study compared the use vitamin D3 (100,000 IU/month) with placebo over 3.3 years in healthy adults from New Zealand with a normal baseline serum 25OHD concentration of 66 nmol/L (26.5 ± 9.0 ng/mL) and a rather high mean calcium intake. Vitamin D supplementation did not reduce the incidence of fractures, falls, cardiovascular disease, or cancer.^26^, ^27^, ^28^ Vitamin D supplementation, however, significantly decreased central blood pressure in subjects with poor vitamin D status at baseline,^29^ and also significantly improved the forced expiratory volume in vitamin‐D‐deficient or even vitamin‐D‐replete subjects with prior chronic obstructive pulmonary disease (COPD) or asthma, or who were (ex)smokers.^30^

The 5‐year Vitamin D and Omega‐3 Trial (VITAL) focused, in part, on vitamin D3 2000 IU/d versus placebo in a study of 25,871 US participants. The participants too were essentially vitamin D replete at baseline with mean serum 25OHD concentrations of 30.8 ±10.0 ng/mL. Vitamin D supplementation did not decrease the incidence of cancer, cardiovascular disease, or overall disease‐specific mortality.^31^ When evaluating the risk of cancer death 2 years after randomization, the odds ratio of cancer death was 0.83 (95% CI, 0.67 to 1.02) in vitamin D supplemented subjects. However, vitamin D supplementation reduced cancer mortality in the subgroup with BMI <25. Therefore, additional data are needed to define the potential long‐term role of vitamin D status on cancer incidence or prognosis, especially in view of the long latency of most cancers. The effects of vitamin D supplementation on diabetes outcome are uncertain or negative. A recent meta‐analysis in patients with type 2 diabetes showed marginal or no effects of vitamin D supplementation.^32^ The large Vitamin D and Type 2 Diabetes (D2d) Study^33^ did not reveal any beneficial effects of vitamin D supplementation (4000 IU/d) for 2.5 years on the progression of prediabetes to full type 2 diabetes, even with achieving rather high final serum 25OHD concentrations (mean values of more than 50 ng/mL). Similarly, the final serum 25OHD concentrations in most other major recent RCTs were well above the optimal concentrations defined by the Endocrine Society, so that it is unlikely that most null results were caused by underdosing. However, in all the major RCTs mentioned above, the baseline serum 25OHD indicated that the large majority of participants were not vitamin D deficient according to the IOM criteria.

These results, which are not consistent with strong preclinical and epidemiologic data, have raised many questions. Vitamin D is a threshold nutrient, comparable to iodine. A threshold nutrient will provide benefits up to a nutritionally sufficient value, above which no further benefit would be expected. Only subjects with poor or marginal status would be expected to benefit from supplementation with a threshold nutrient. This concept, as valid as it surely is, was not followed in the design of these large clinical trials. It would appear that the baseline serum 25OHD concentration was sufficient in the majority of subjects in the ViDA, VITAL, or D2d trials. The negative results of these trials, therefore, should not have been totally unexpected. Moreover, the ViDA trial used high intermittent doses that are now considered of questionable efficacy^34^ or safety,^35^resulting in mean serum 25OHD levels well above the recommended IOM upper limit (50 ng/mL). Despite the apparently negative data from these trials, the effects of vitamin D on the immune system have been reported to be more positive. A recent meta‐analysis of RCTs shows a protective effect of vitamin D on the incidence of acute respiratory infections.^34^ The effect was greater in participants with a baseline serum 25OHD lower than 25 nmol/L (10 ng/mL). The effect was not seen with bolus doses of vitamin D, but was present in those who received daily or weekly doses. Two other meta‐analyses show that vitamin D may protect against acute exacerbations of asthma and COPD.^36^, ^37^

For some extraskeletal outcomes, RCTs may be hardly realistic because of the necessarily high numbers of participants and the required long duration. Diseases such as cancer and multiple sclerosis may develop over a decade or more. To answer the many remaining questions, it would be wise to plan individual participant data meta‐analyses of the major RCTs (whether already published or in progress) and preferably after recalibration of the serum 25OHD concentrations by appropriate methods to assure uniform accuracy. A MR study may offer an alternative. In a MR study, groups are defined by several single nucleotide polymorphisms (SNPs) that influence the serum 25OHD concentration. Subjects with genetically predicted lower serum 25OHD concentrations have been found in all three large MR studies to have a significantly higher risk to develop multiple sclerosis; one such study confirmed that for another major autoimmune disease, type 1 diabetes mellitus.^5^ MR studies on vitamin D are handicapped by the relatively low predictive value of SNPs (about 5% in the large studies cited in ref 5) so that positive results suggest that even small differences from very early in life onwards may have extraskeletal health consequences. The low predictive value for variations in serum 25OHD, however, also implies that null results do not exclude a positive link between vitamin D status and other outcomes. Newer genetic studies now would allow the study of more polymorphisms that, when combined, are able to predict nearly 10% of the lifetime variation of serum 25OHD.

Based on these new data and insights, what can we recommend to clinicians? First, rickets still is highly prevalent in the Middle East and in some Asian countries.^38^ It is rare in Europe, North America, and Australia‐New Zealand, except in infants and children from vitamin‐D‐deficient mothers and those who do not receive vitamin D supplements.^39^, ^40^ Over the last decade, an increase in the incidence of rickets in the United Kingdom was observed because of a lower rate of vitamin D supplementation in neonates.^41^ The same was observed in Scandinavian countries. On the other hand, a campaign of providing free vitamin D supplements in Turkey has reduced the incidence of rickets from 6% to 0.1% in a few years.^42^ It should be feasible to eradicate rickets by worldwide campaigns of vitamin D supplementation during the first, or preferably the first 3 years of life. The WHO should take the lead in such eradication programs.

Vitamin D supplementation in pregnancy still is a controversial issue. An umbrella review of systematic reviews and meta‐analyses of observational studies and RCTs concluded that an association between serum 25OHD concentrations and birthweight, dental caries in children, and maternal 25OHD concentrations at term is probable, but better designed trials are necessary.^12^ Another meta‐analysis showed a higher mean birthweight and a trend to less gestational diabetes.^43^ A recent clinical trial in Iran showed a decrease of preeclampsia, gestational diabetes, and preterm delivery in the vitamin D group.^44^ Two clinical trials addressed the effect of vitamin D supplementation during pregnancy on wheezing or asthma in the offspring during the first 3 years. In one of these studies, vitamin D supplementation decreased the number of episodes of troublesome lung symptoms;^45^ in the other, vitamin D supplementation resulted in a borderline decrease of asthma or recurrent wheezing.^46^ A Cochrane meta‐analysis concluded that vitamin D supplementation may reduce the risk of preeclampsia, low birthweight, and preterm birth, but more rigorous studies are needed.^47^ Taken together, the evidence for vitamin D supplementation during pregnancy is not overwhelming. However, vitamin D deficiency is very common in pregnancy and even more in risk groups such as non‐Western immigrants,^48^ and the potential benefit of vitamin D supplementation may be great for mother and child.

Vitamin D deficiency is very prevalent in the Western world. In Europe, vitamin D deficiency (serum 25OHD <50 nmol/L) was observed in 40% of participants in 12 cohort studies assessed with standardized 25OHD measurements according to the Vitamin D Standardization Program.^49^ Severe vitamin D deficiency (serum 25OHD <25 nmol/L or 10 ng/mL) was seen in 12.5%. A systematic world review of vitamin D status showed that mean serum 25OHD was lower than 50 nmol/L in one‐third of all studies.^50^ Risk groups are the elderly and non‐Western immigrants.^48^, ^51^ A reasonable approach, suggested by a working group of the European Calcified Tissue Society, is to implement programs to stimulate fortification of foods with vitamin D, eg, fortification of milk with 400 IU (10 μg) per liter.^52^ This or a similar approach in line with local food habits would increase vitamin D status throughout the world. Such a fortification approach was very successful in Finland, where the prevalence of vitamin D deficiency among the population decreased from 50% in 2000 to 6% in 2011.^53^

Regrettably, recent publications and opinion articles on this subject have not been ideally balanced. Vitamin D is neither a panacea nor the fountain of youth, despite the temptation among some aficionados to believe it to be. Vitamin D cannot cure most chronic diseases. However, it is very important for optimal skeletal development, to prevent fractures in frail elderly, and for the development and maintenance of the immune system. We should encourage vitamin D supplementation in moderate doses for all who need it, especially infants, young children, pregnant women, immigrants with dark skin living in moderate climates, and (frail) older persons. Clearly, any individual with a vitamin D deficiency should also be treated. Vitamin D supplementation should always be associated with age‐related optimal calcium intake. Although some people take more vitamin D than they need, far too many are not taking enough to secure adequate nutritional status.

## Disclosures

All authors have nothing to disclose.
